# *CCNB2*, *TOP2A*, and *ASPM* Reflect the Prognosis of Hepatocellular Carcinoma, as Determined by Weighted Gene Coexpression Network Analysis

**DOI:** 10.1155/2020/4612158

**Published:** 2020-06-23

**Authors:** Yuping Zeng, He He, Yu Zhang, Xia Wang, Lidan Yang, Zhenmei An

**Affiliations:** ^1^Department of Laboratory Medicine, West China Hospital, Sichuan University, Chengdu, China; ^2^Department of Endocrine and Metabolism, West China Hospital, Sichuan University, Chengdu, China

## Abstract

**Background:**

Hepatocellular carcinoma (HCC) is characterized by increased mortality and poor prognosis. We aimed to identify potential prognostic markers by weighted gene coexpression network analysis (WGCNA), to assist clinical outcome prediction and improve treatment decisions for HCC patients.

**Methods:**

Prognosis-related gene modules were first established by WGCNA. Venn diagrams obtained intersection genes of module genes and differentially expressed genes. The Kaplan-Meier overall survival curves and disease-free survival curves of intersection genes were further analyzed on the Gene Expression Profiling Interactive Analysis website. Chi-square tests were performed to explore the associations between prognostic gene expressions and clinicopathological features.

**Results:**

*CCNB2*, *TOP2A*, and *ASPM* were identified as both prognosis-related genes and differentially expressed genes. *TOP2A* (HR: 1.7, *P* = 0.003) and *ASPM* (HR: 1.8, *P* < 0.001) exhibited a significant difference between the high- and low-expression groups in the overall survival analysis, while *CCNB2* (HR: 1.4, *P* = 0.052) was not statistically significant. *CCNB2* (HR: 1.5, *P* = 0.006), *TOP2A* (HR: 1.7, *P* < 0.001), and *ASPM* (HR: 1.6, *P* = 0.003) were all statistically significant in the disease-free survival analysis. All three genes were significantly associated with race and fetoprotein values (*P* < 0.05). *CCNB2* expression was associated with tumor stage (*P* = 0.01), and *ASPM* expression was associated with new tumor events (*P* = 0.03).

**Conclusion:**

Overexpression of *CCNB2, TOP2A*, and *ASPM* are associated with poor prognosis, and these genes could serve as potential prognostic markers and therapeutic targets for HCC.

## 1. Introduction

Hepatocellular carcinoma (HCC) is the third most common cause of cancer mortality worldwide [1]. The mechanisms by which hepatitis B virus, hepatitis C virus, alcohol, fatty liver disease, and other environmental factors, such as aflatoxin, cause liver cancer remain unclear. However, advances in genomics provide essential information about tumor initiation and progression [2]. The complex genetic background of HCC makes current clinical staging methods, including the Barcelona Clinic Liver Cancer algorithm or the tumor node metastasis (TNM) staging, insufficient to predict patient prognosis. Indeed, patients with the same HCC stage may have significantly different outcomes. Besides, the high recurrence and metastasis rates after ablation or surgical resection lead to a low survival rate in patients with HCC. These outcomes present an urgent requirement for improved prognostic estimates other than staging [3]. To date, thousands of HCC genomes have been sequenced globally, and most driver gene mutations, structural variants, fusion genes, copy number alterations, and viral integration events have been established [4]. This vast amount of new data provides an opportunity to understand the molecular basis of HCC better [5]. However, to utilize this scientific information, knowledge of the available database resources and bioinformatics tools are indispensable. To improve the treatment decisions and the overall survival of patients with HCC, we summarized the currently available databases supporting HCC research to aid in the identification of potential prognostic markers of clinical outcome prediction.

A coexpression analysis is an efficient method to describe free-scale gene coexpression networks. The weighted gene coexpression network analysis (WGCNA), an algorithm based on large-scale datasets and modules of highly correlated genes, was used to explore associations between gene sets and clinical features and to identify candidate biomarkers [6, 7]. This approach has been successfully applied in multiple tumors, such as clear cell renal cell carcinoma [8], glioblastoma [6], pancreatic carcinoma [9], adrenocortical carcinoma [10], breast cancer [11], and HCC [12, 13]. We mined prognostic markers by constructing a coexpression network and performing differential gene analysis and survival analysis to verify their prognostic values.

## 2. Materials and Methods

### 2.1. Data Sources

HCC-related gene expression profiles were downloaded from Gene Expression Omnibus (GEO) datasets (https://www.ncbi.nlm.nih.gov/geo/).The search details used were as follows: (“2010”(UDAT): “3000”(UDAT)) and (((“carcinoma, hepatocellular”(MeSH terms) or hepatocellular carcinoma (all fields)) and (“liver neoplasms”(MeSH terms) or liver cancer (all fields)) and (“mortality”(subheading) or “survival”(MeSH terms) or survival (all fields))) and “Homo sapiens”(porgn) and “Expression profiling by array”(filter)) and “Expression profiling by array”(filter). Four datasets contained HCC and paracancerous nontumor tissues, and samples over 30 were included for our analysis. GSE54236 [14] was utilized for WGCNA analysis because there were clinical features such as survival time in the dataset. GSE60502 [15], GSE64041 [16], and GSE45114 [17] were used for differential expression gene (DEG) analysis to obtain reliable DEGs. Standardized series matrix file and annotation platform were extracted through the R package “GEOquery” [18]. The data uniformity between samples was judged by boxplot and log2 conversion was performed to standardize datasets if necessary.

### 2.2. WGCNA Analysis

To identify hub genes associated with phenotypes, we analyzed GSE54236 by the R package **“**WGCNA” as follows [19, 20]. (1) Data procession: the median absolute deviation (MAD) of each gene expression between samples was calculated. We selected genes with MAD greater than the 70% quantile interval of the MAD of all genes and greater than 0.01 for the subsequent analysis. Missing values were checked by the “goodSamplesGenes” function, and outlier samples were identified by the “hclust” function to eliminate possible sample interference. (2) Network construction: soft power was determined with a threshold of 0.85 by “pickSoftThreshold” function to make the constructed network in line with a scale-free network. Then, we constructed the network by “blockwiseModules” function and showed each module by a hierarchical clustering tree. (3) Modules related to phenotypes: To explore phenotype-related modules, we analyzed the associations between modules and phenotypes. We calculated the correlations of modules and genes (module membership), and phenotypes and genes (gene significance) by the Pearson correlation analysis. Genes highly associated with phenotypes in modules, with correlation coefficients > 0.5 and *P* < 0.05 were selected for further analysis. (4) Hub genes identified: the topological overlap matrix was calculated from module genes of interest, and hub nodes with an edge-adjacency threshold of 0.2 were exported to visualize in Cytoscape (version 3.7.2) by “exportNetworkToCytoscape” function.

### 2.3. Pathway Enrichment Analysis

We performed Gene Ontology (GO), including biological processes, cellular components, and molecular functions, and the Kyoto Encyclopedia of Genes and Genomes (KEGG) enrichment analysis of module genes of interest by the “clusterProfiler” package [21]. Next, the top 10 most significant enrichment pathways were graphically displayed. Moreover, protein-protein interaction (PPI) analysis was performed on the Metascape website (http://metascape.org/gp/index.html#/main/step1), which determined the PPI network, Molecular Complex Detection (MCODE) components, and the top three enrichment pathways of each component.

### 2.4. DEG Analysis

We analyzed DEGs of GSE60502, GSE64041, and GSE45114 between HCC and paracancerous nontumor tissues by the “limma” [22] package. ∣Log2 fold change | >1.5 and adjusted *P* values (Benjaminiand-Hochberg adjustment) < 0.05 were set as the screening cutoff for DEGs. The intersection of WGCNA phenotype-related module genes and DEGs was obtained and visualized in Venn diagrams (http://bioinfogp.cnb.csic.es/tools/venny/index.html), representing both prognosis-related and differentially expressed genes.

### 2.5. Survival Analysis

We performed the Kaplan-Meier overall survival curves and disease-free survival curves of intersection genes on the Gene Expression Profiling Interactive Analysis (GEPIA) website [23], a web-based tool for analyzing Cancer Genome Atlas (TCGA) and Genotype-Tissue Expression (GTEx) project data from tumors and normal samples.

### 2.6. Correlation Analysis of Clinicopathological Features

To further explore the associations between prognosis gene expressions and clinicopathological features, RNA-seq-counts of the TCGA liver cancer (*n* = 424) and phenotypes (*n* = 469) were downloaded from the UCSC Xena (https://xenabrowser.net/datapages/). Genes were divided into high- and low-expression groups, according to the median of gene expressions. We compared the differences between the two groups in clinicopathological features by Chi-square tests. All analyses were performed by R (version 3.6.0). Bilateral *P* values less than 0.05 were considered statistically significant.

## 3. Results

### 3.1. Weighted Coexpression Network Construction and Key Module Identification

**Figure 1:**
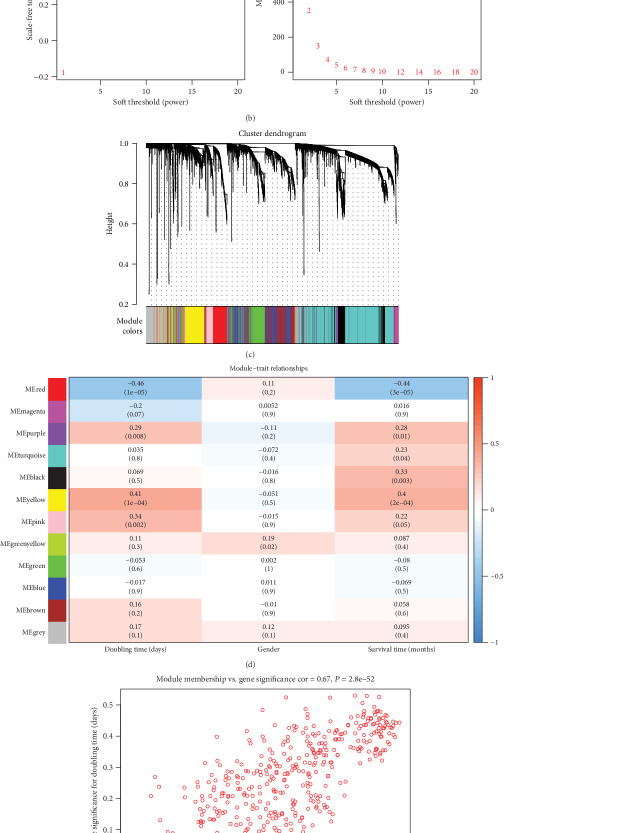
Weighted gene coexpression network analysis of GSE54236. (a) Sample clustering tree to detect outliers. (b) Identification of soft power with a threshold of 0.85. (c) Hierarchical clustering tree of each module. Different colors represent different modules. (d) Correlations of modules and doubling time and survival time. The correlation coefficient varied from -1 (blue) to 1 (red) and *P* was annotated. (e) Correlations between gene-module and gene-survival time. (f) Correlations between gene-module and gene-doubling time.

To mine coexpression module genes for HCC, the top 30% MAD of all genes in GSE54236 were extracted, which provided 5,612 genes for further analysis. No outlier samples were identified in the sample clustering tree, so no samples were removed from the analysis (Figure 1(a)). Next, we constructed the gene-gene similarity network. We set the soft power as 7 to ensure a scale-free network (Figure 1(b)). By the “blockwiseModules” function, we divided the network into 11 modules with similar gene expressions (Figure 1(c)). Then, we analyzed the relationships between modules and phenotypes. Results showed that the red module had a significant negative correlation with doubling time and survival time (Figure 1(d)). Figures 1(e) and 1(f) further proved the associations between the red module genes and doubling time and survival time with correlation coefficients > 0.5 and *P* < 0.001. Finally, we identified 153 hub genes in the red module as key genes by Cytoscape.

### 3.2. Gene Function and Pathway Enrichment Analyses

**Figure 2:**
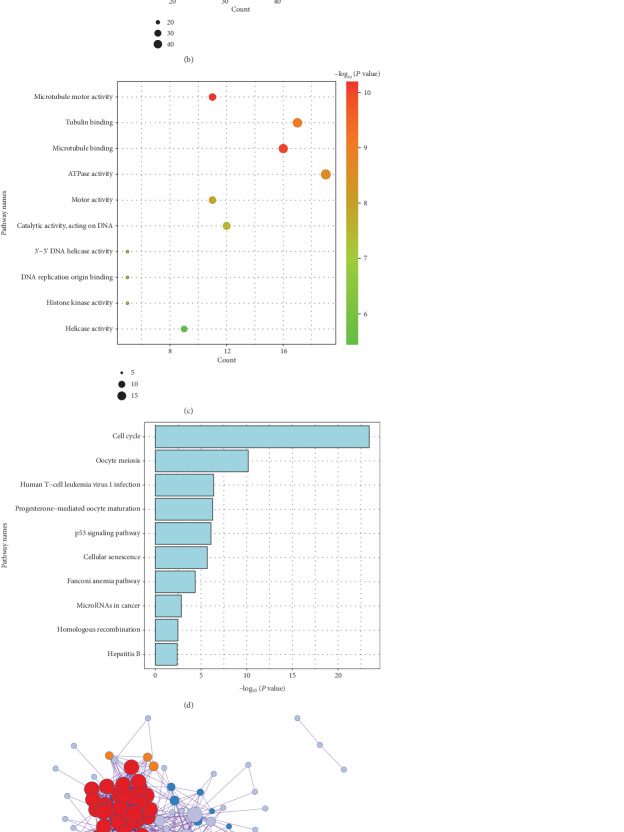
Enrichment analysis of red module genes. (a) Biological process analysis. (b) Cellular component analysis. (c) Molecular function analysis. (d) Kyoto Encyclopedia of Genes and Genomes (KEGG) analysis. (e) Protein-protein interaction (PPI) network. (f) Molecular Complex Detection (MCODE) analysis.

To further understand the biological functions and signaling pathways in which red module genes participate, we performed enrichment analysis for 153 hub genes by GO and KEGG. Biological processes were mainly enriched in chromosome segregation, nuclear division, and organelle fission (Figure 2(a)). Associated cell components were mainly present in the chromosomal region, chromosome, centromeric region, and condensed chromosome (Figure 2(b)). Molecular functions occurred in microtubule motor activity, tubulin binding, and microtubule activity (Figure 2(c)). KEGG pathways were considerably enriched in the cell cycle and oocyte meiosis (Figure 2(d)). PPI and MCODE analyses revealed that red module genes were divided into five components (Figures 2(e) and 2(f)). The top three enrichment pathways for each component were shown in Supplementary Table 1. These genes mainly participated in the cell cycle and divisions.

### 3.3. Identification of Intersection Genes

**Figure 3:**
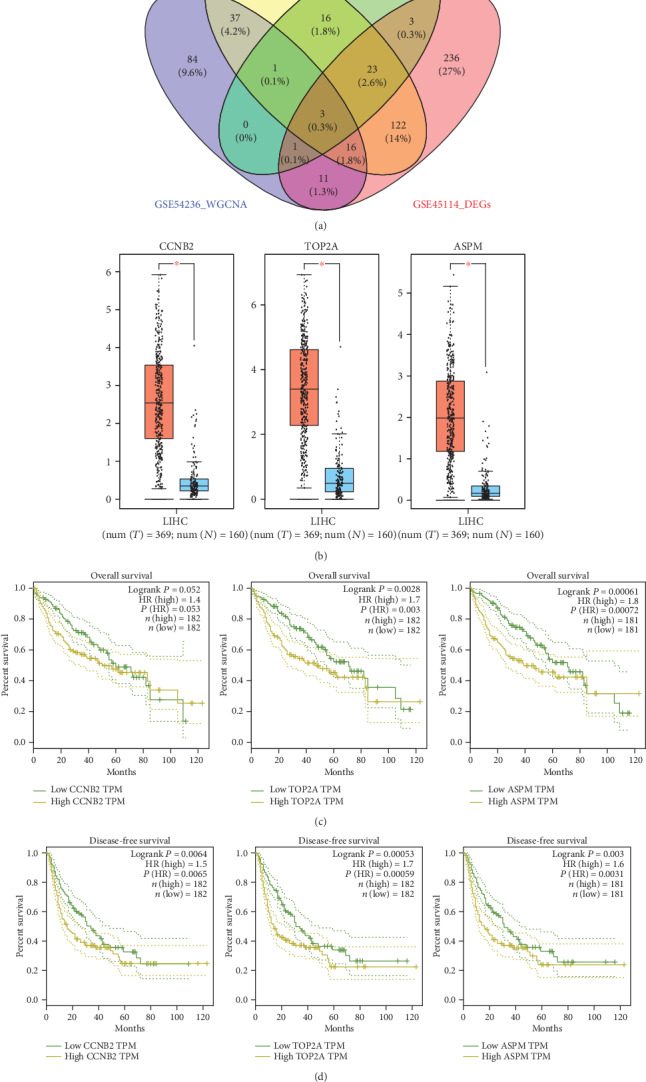
Survival analysis of *CCNB2*, *TOP2A*, and *ASPM*. (a) Venn diagrams of red module genes in weighted gene coexpression network analysis (WGCNA) and differentially expressed genes (DEGs). (b) Expression levels. (c) Overall survival curves. (d) Disease-free survival curves of *CCNB2*, *TOP2A*, and *ASPM*.

We explored the intersection genes both related to prognosis and differentially expressed in tumors and paracancerous nontumor tissues by the Venn diagram. There were 510, 75, and 415 DEGs for GSE60502, GSE64041, and GSE45114, respectively. We found three intersection genes (*CCNB2*, *TOP2A*, and *ASPM*) which were both red module genes and DEGs in the Venn diagram (Figure 3(a)).

### 3.4. Identification and Validation of Hub Genes

To further validate hub genes, we analyzed expression levels (Figure 3(b)), overall survival curves (Figure 3(c)), and disease-free survival curves (Figure 3(d)) of *CCNB2*, *TOP2A*, and *ASPM* on the GEPIA website. All three genes were differentially expressed in HCC and normal tissues with *P* < 0.01. Genes were divided into high- and low-expression groups based on the median gene expression levels. The differences between the two groups were compared by the logrank test. *TOP2A* (HR: 1.7, *P* = 0.003) and *ASPM* (HR: 1.8, *P* < 0.001) had a statistical significance between the two groups in the overall survival analysis, while *CCNB2* (HR: 1.4, *P* = 0.052) was not statistically significant. *CCNB2* (HR: 1.5, *P* = 0.006), *TOP2A* (HR: 1.7, *P* < 0.001), and *ASPM* (HR: 1.6, *P* = 0.003) were statistically significant in the disease-free survival analysis.

### 3.5. Associations between Gene Expressions and Clinicopathological Features

To explore the associations between *CCNB2*, *TOP2A*, *ASPM*, and clinicopathological features, we divided three genes into the low- and high-expression groups according to the median of gene expressions in the TCGA. Through Chi-square tests, we found that all three genes were significantly associated with race and fetoprotein (AFP) values (*P* < 0.05). *CCNB2* was significantly associated with tumor stage (*P* = 0.01), while there was no significant association between *TOP2A* or *ASPM* and tumor stage. Moreover, a significant association between *ASPM* and new tumor events was observed (*P* = 0.03), as shown in Table 1. We did not found significant associations between *CCNB2*, *TOP2A*, *ASPM*, and BMI, adjacent hepatic tissue inflammation, fibrosis Ishak score, residual tumor, vascular tumor, and Child-Pugh classification, as shown in Supplementary Table 2.

## 4. Discussion

Clinical prognostic information is limited for patients of HCC. Several staging systems that attempt to predict patient prognosis and guide treatment modality have been proposed for HCC. However, the clinical features of these systems have drawbacks concerning the efficacy of treatment guidance and prognostic accuracy. Staging systems based on clinical features lack universal applicability, having been designed using data mostly acquired from a Western population or by being too rigid in suggesting therapy for some problematic prognostic factors [24]. Therefore, it is necessary to identify prognostic markers for patients with HCC. Patients with HCC present with features of high heterogeneity, metastasis, recurrence, and mortality and provide potential targets for prognosis prediction and treatment monitoring [25]. On the other hand, there have been very few reports detailing data mining by combining coexpressed and differentially expressed genes to explore HCC prognostic markers.

In this study, we explored genes associated with HCC prognosis using WGCNA, a method more frequently used to investigate phenotype-related coexpression module genes than to analyze DEGs. We identified 153 hub genes in the red module that were significantly negatively associated with doubling time and survival time, symbolizing a poor prognosis. Besides, the strong correlations of modules and genes and phenotypes and genes further proved the robust prognostic efficacy of red module genes. Second, gene function and pathway enrichment analyses indicated that red module genes were mainly engaged in the cell division and proliferation of HCC. DEGs were mined by combining GSE60502, GSE64041, and GSE45114, making results more credible. Further combining overall and disease-free survival analysis using the GEPIA website, we identified that *TOP2A*, *ASPM*, and *CCNB2* were promising prognostic markers and potential therapeutic targets for HCC. It is worth noting that the overall survival analysis was based on patient all-cause mortality, and disease-free survival analysis was based on disease progression or death. Therefore, overall survival analysis endpoints were optimal observation endpoints without subjective judgment bias. In our study, both overall and disease-free survival analyses were evaluated by public databases.

We further investigated associations between *TOP2A*, *ASPM*, *CCNB2*, and clinicopathological features by Chi-square tests. We found significant associations between three prognostic genes and AFP levels; therefore, we speculated that these three genes were potential markers of the development and progression of HCC. However, whether these genes might become favorable diagnostic markers for HCC and specific mechanisms required further experimental verification. Importantly, high *CCNB2* expression was strongly associated with tumor stage (*P* = 0.01), and high *ASPM* expression was associated with new tumor events (*P* = 0.03). These findings indicated that the overexpression of *CCNB2* and *ASPM* accelerated the possibility of HCC deterioration and metastasis, suggesting a worse prognosis for HCC patients; hence, more aggressively integrated treatment and assessment were needed when these genes were found to be overexpressed.

*TOP2A*, DNA topoisomerase II alpha encoding a DNA topoisomerase, regulates the topologic states of DNA and controls tumor cell response. Wong et al. [26] showed that *TOP2A* overexpression in HCC tumors, relative to adjacent nontumors, correlated with histological grading, microvascular invasion, tumor deterioration, shorter survival, and chemoresistance, which was consistent with our clinicopathological features correlation analysis [27]. *TOP2A* targeted therapeutic drugs, such as doxorubicin, were suggested when *TOP2A* was overexpressed in HCC. Overexpression of microRNA-23a potentiated the response of HCC to the *TOP2A* targeted drugs etoposide and doxorubicin *in vivo* and *in vitro*, while suppressing another topoisomerase, *TOP1*, but not altering *TOP2A* expression levels [28]. Another study showed that *TOP2A* was targeted for proteasomal degradation by histone deacetylase inhibitors by activating casein kinase 2*α* and GSK3*β* double phosphorylation, highlighting a novel potential mechanism of HCC treatment [29]. The significance of *TOP2A* in tumor aggressiveness and chemoresistance indicate that the mechanisms through which it functions require further exploration.

*ASPM* encodes an abnormal spindle-like, microcephaly-associated protein that participates in mitosis and is reported to be a recurrence, invasion, metastasis, and prognostic marker for HCC. However, the specific molecular mechanism and pathways through which ASPM function have not been reported [30, 31]. *CCNB2* encodes cyclin B2, the cell cycle mediated by transforming growth factor *β*, which, according to the GEPIA website, did not have a statistically significant influence on overall survival. However, we further analyzed the influence on *CCNB2* on the UALCAN website [32] (http://ualcan.path.uab.edu/index.html) and found a statistical significance with *P* < 0.001. The variation in these results could be attributed to the different sample sizes and sequencing databases. A review of the literature revealed that *CCNB2* expression is increased in HCC tissues compared to adjacent nontumor tissues [33], and *CCNB2* is a target molecule following knockdown of *XPOT* [34], *TPX2* [35], *KPNA2* [36], and *TMEM9* [37]. The prognostic value and target therapy of the hub genes for HCC patients remain to be confirmed by further clinical studies.

There were some limitations in our study. All of these sequencing data were from tissue samples and may not be suitable for unresectable patients whose liver tissues may be unavailable. Given that bioinformatics predictions are obtained in silico, further experimental validation is required. Additionally, we analyzed expression profiling by the array in the GEO datasets and concluded that *TOP2A*, *ASPM*, and *CCNB2* were promising prognostic markers and potential therapeutic targets. However, other prognostic markers, such as methylation, microRNA, lncRNA, and circRNA, should be explored, and the data should be integrated with the findings of the present study to propose a new treatment algorithm that incorporates these markers in determining how aggressive is the treatment approach.

## Figures and Tables

**Table 1:** Associations between *CCNB2*, *TOP2A*, and *ASPM* expressions and clinicopathological features.

*Variables*		*CCNB2*				*TOP2A*				*ASPM*			
		Low (*n*)	High (*n*)	*χ* ^2^	*P*	Low (*n*)	High (*n*)	*χ* ^2^	*P*	Low (*n*)	High (*n*)	*χ* ^2^	*P*
Age (years)	<50	29	39	1.41	0.24	30	38	0.84	0.36	31	37	0.42	0.52
	≥50	152	143			151	144			150	145		

Gender	Female	51	67	2.70	0.10	50	68	3.49	0.06	54	64	0.95	0.33
	Male	130	115			131	114			127	118		

Race	Asian	65	90	6.26	*0.01*	63	92	8.56	*0.003*	66	89	5.24	*0.02*
	Non-Asian	116	92			118	90			115	93		

New tumor events	No	108	91	3.05	0.08	104	95	0.81	0.37	110	89	4.70	*0.03*
	Yes	73	91			77	87			71	93		

Tumor stage	I-II	149	129	6.00	*0.01*	147	131	3.82	0.05	146	132	2.91	0.09
	III-IV	32	53			34	51			35	50		

Fetoprotein (ng/ml)	<500	125	91	17.07	*<0.001*	121	95	10.19	*0.001*	119	97	9.39	*0.002*
	≥500	16	44			19	41			19	41		

## Data Availability

The data used to support the findings of this study are available from the corresponding author upon request.

## Supplementary Materials

Supplementary Materials Supplementary Table 1: the top three enrichment pathways of five MCODE components. Supplementary Table 2: association between CCNB2, TOP2A, and ASPM expressions and other clinical features.
