# Supplementary material for: CCNB2, TOP2A, and ASPM Reflect the Prognosis of Hepatocellular Carcinoma, as Determined by Weighted Gene Coexpression Network Analysis
Source: Biomed Res Int. 2020 Jun 23;2020:4612158. doi: 10.1155/2020/4612158 (PMC7333053; doi:10.1155/2020/4612158)
Supplement: Supplementary Materials — Supplementary Table 1: the top three enrichment pathways of five MCODE components. Supplementary Table 2: association between CCNB2, TOP2A, and ASPM expressions and other clinical features. [file 4612158.f1.doc]

**Supplementary Table 1. The top three enrichment pathways of five MCODE components.**

| **MCODES** | **GO** | **Descriptions** | **Log10(*P*)** |
| --- | --- | --- | --- |
| **MCODE_1** | R-HSA-2500257 | Resolution of Sister Chromatid Cohesion | -72.5 |
|  | R-HSA-68877 | Mitotic Prometaphase | -65.6 |
|  | R-HSA-141444 | Amplification of signal from unattached kinetochores via a MAD2 inhibitory signal | -62.1 |
| **MCODE_2** | R-HSA-5685942 | HDR through Homologous Recombination (HRR) | -12.8 |
|  | R-HSA-1640170 | Cell Cycle | -12.5 |
|  | R-HSA-5693554 | Resolution of D-loop Structures through Synthesis-Dependent Strand Annealing (SDSA) | -12.4 |
| **MCODE_3** | R-HSA-68962 | Activation of the pre-replicative complex | -15.5 |
|  | R-HSA-176187 | Activation of ATR in response to replication stress | -15.2 |
|  | GO:0044772 | mitotic cell cycle phase transition | -14.9 |
| **MCODE_4** | R-HSA-8854518 | AURKA Activation by TPX2 | -10.1 |
|  | GO:0010389 | regulation of G2/M transition of mitotic cell cycle | -8.8 |
|  | GO:1902749 | regulation of cell cycle G2/M phase transition | -8.6 |
| **MCODE_5** | GO:0061641 | CENP-A containing chromatin organization | -8.2 |
|  | GO:0034080 | CENP-A containing nucleosome assembly | -8.2 |
|  | GO:0031055 | chromatin remodeling at centromere | -8.2 |

GO: Gene Ontology; MCODE: Molecular Complex Detection.

**Supplementary Table 2. Association between *CCNB2*, *TOP2A*, *ASPM* expressions and other clinical features.**

| **Variables** | | ***CCNB2*** | | | |  | ***TOP2A*** | | | |  | ***ASPM*** | | | |
| --- | --- | --- | --- | --- | --- | --- | --- | --- | --- | --- | --- | --- | --- | --- | --- |
|  |  | ***Low***  ***(n)*** | ***High***  ***(n)*** | ***χ^2^*** | ***P*** |  | ***Low***  ***(n)*** | ***High***  ***(n)*** | ***χ^2^*** | ***P*** |  | ***Low***  ***(n)*** | ***High***  ***(n)*** | ***χ^2^*** | ***P*** |
| **BMI** | <25 | 81 | 94 | 1.74 | 0.187 |  | 80 | 95 | 2.03 | 0.155 |  | 90 | 85 | 0.19 | 0.660 |
|  | ≥25 | 85 | 72 |  |  |  | 85 | 72 |  |  |  | 76 | 81 |  |  |
| **Adjacent hepatic tissue inflammation** | No | 118 | 130 | 1.35 | 0.245 |  | 121 | 127 | 0.24 | 0.626 |  | 127 | 124 | 0.00 | 1.000 |
|  | Yes | 63 | 52 |  |  |  | 60 | 55 |  |  |  | 57 | 58 |  |  |
| **Fibrosis ishak score** | 0-4 | 139 | 147 | 0.64 | 0.425 |  | 145 | 141 | 0.24 | 0.627 |  | 144 | 142 | 0.05 | 0.818 |
|  | 5-6 | 42 | 35 |  |  |  | 36 | 41 |  |  |  | 37 | 40 |  |  |
| **Residual tumor** | R0 | 165 | 162 | 0.26 | 0.611 |  | 163 | 164 | 0.00 | 1.000 |  | 165 | 162 | 0.26 | 0.611 |
|  | R1-X | 16 | 20 |  |  |  | 18 | 18 |  |  |  | 16 | 20 |  |  |
| **Vascular tumor** | No | 131 | 128 | 0.10 | 0.753 |  | 130 | 129 | 0.01 | 0.934 |  | 133 | 126 | 0.61 | 0.436 |
|  | Yes | 50 | 54 |  |  |  | 51 | 53 |  |  |  | 48 | 56 |  |  |
| **Child pugh classification** | A | 168 | 173 | 0.45 | 0.501 |  | 169 | 172 | 0.05 | 0.816 |  | 170 | 171 | 0.00 | 1.000 |
|  | B-C | 13 | 9 |  |  |  | 12 | 10 |  |  |  | 11 | 11 |  |  |
